# Functions of Cationic Host Defense Peptides in Immunity

**DOI:** 10.3390/ph9030040

**Published:** 2016-07-04

**Authors:** Mahadevappa Hemshekhar, Vidyanand Anaparti, Neeloffer Mookherjee

**Affiliations:** Manitoba Centre for Proteomics and Systems Biology, Departments of Internal Medicine and Immunology, University of Manitoba, Winnipeg, MB R3E3P4, Canada; Hem.Mahadevappa@umanitoba.ca (M.H.); Vidyanand.Anaparti@umanitoba.ca (V.A.)

**Keywords:** cationic host defense peptides, antimicrobial peptides, immunomodulation, inflammation

## Abstract

Cationic host defense peptides are a widely distributed family of immunomodulatory molecules with antimicrobial properties. The biological functions of these peptides include the ability to influence innate and adaptive immunity for efficient resolution of infections and simultaneous modulation of inflammatory responses. This unique dual bioactivity of controlling infections and inflammation has gained substantial attention in the last three decades and consequent interest in the development of these peptide mimics as immunomodulatory therapeutic candidates. In this review, we summarize the current literature on the wide range of functions of cationic host defense peptides in the context of the mammalian immune system.

## 1. Introduction

Cationic host defense peptides (CHDPs) are immunomodulatory molecules that have evolved to provide broad range of protection against a variety of pathogenic microbes. Early research in the field focused primarily on the antimicrobial functions of CHDPs, consequently these molecules are also known as antimicrobial peptides. However, studies have shown that certain microbial pathogens can be resistant to the direct microbicidal activity of CHDPs at physiological concentrations [1,2]. Moreover, it has been demonstrated that the direct antimicrobial property of certain CHDPs are often compromised in the presence of physiological salt concentrations and specific host factors [3,4], yet these peptides are essential for the protection against pathogens [5,6]. This highlights the role of CHDPs in immunity, which is to enhance microbial clearance and maintain immune homeostasis [7,8]. Therefore, the term CHDPs has now been adopted to encompass the antimicrobial, immunological and other biological functions of these molecules that have been defined over the last three decades [7,9,10]. A wide range of species from microbes to plants and mammals express CHDPs. Cationic peptides with antimicrobial activity were originally identified from lysosomal fractions of leukocytes from guinea-pigs in the 1960s [11]. Subsequently, Lehrer and Selsted purified cysteine-linked antimicrobial peptides from rabbit leukocytes in the 1980s [12,13]. Subsequent research has led to the characterization of more than 2600 cationic peptides from a wide variety of organisms ranging from microbes to plants to humans.

The two most well characterized families of CHDPs in mammals are cathelicidins and defensins. We will focus on these two families of mammalian CHDPs in this review. These CHDPs are expressed by various cell types including macrophages, polymorphonuclear leukocytes such as neutrophils, mast cells, stromal bone marrow and mucosal epithelial cells. CHDPs are small amphipathic peptides typically 12–50 amino acids in length, with a net positive charge ranging from +2 to +9 due to an abundance of basic amino acids such as lysine and arginine [14,15,16]. CHDPs are derived from immature precursor proteins that subsequently undergo proteolytic cleavage to render the biologically active mature peptides. CHDPs have diverse sequences and structures, and are broadly classified into two categories, α-helical peptides and β-sheet class peptides, based on their length, structure and the presence of disulfide linkages.

A prominent example of amphipathic α-helical CHDPs is the cathelicidin family of peptides [17,18]. Cathelicidins are comprised of a highly conserved 14 kDa N-terminal cathelin-like pro-domain, followed by a signal peptide and a C-terminal ‘mature’ peptide region. The sole human cathelicidin is expressed as an 18 kDa precursor pro-protein called hCAP18, which is subsequently cleaved to the well characterized, biologically active, 37 amino acid cationic peptide LL-37 [19]. This can be subsequently cleaved to generate smaller peptides that have been identified from epithelial surfaces [20]. LL-37 is expressed constitutively or induced by the presence of infections and/or inflammatory stimuli, in both immune and structural cells, e.g., secretory granules of neutrophils, mast cells, B-cells, T-cells, monocytes, natural killer cells, keratinocytes, squamous and airway epithelial cells [21,22]. Additionally, epithelial cells lining the upper digestive tract, salivary glands, sweat glands, small intestine, epididymis, testis, vagina and cervix also express LL-37 [5,19,23]. A recent study has also demonstrated that LL-37 can be expressed by adipocytes [24]. Structurally, LL-37 is devoid of disulphide bonds and acquires an α-helical conformation when interacting with lipid bilayers [21,22]. Solid-state NMR studies and CD spectroscopy suggest that α-helical LL-37 can also form tetramers or higher oligomers in solution, rendering the peptide cytotoxic at higher concentrations. Extensive solid-state NMR studies have elucidated the conformation, orientation, dynamics and the oligomeric nature of LL-37 in membranes [25].

Amongst the β-sheet class of CHDPs, the mammalian defensins are the best characterized. These are 30–45 amino acid long cyclic peptides that are rich in cysteine, arginine and aromatic amino acid residues and contain cysteine disulphide bridges. Defensins are categorized further as α, β and θ-defensins [17,18,26,27]. The α-defensins were identified from azurophilic neutrophil granules of primates, and are also known as human neutrophil peptides (HNP). There are four different types of HNPs (HNP1-4), of these HNP1-3 are highly homologous, varying by one amino acid at the amino terminus. Thus, it is not surprising that HNP1-3 exhibit an exceptionally high degree of sequence and functional similarity. A wide range of cells and tissues such as neutrophils, peripheral blood mononuclear cells, bone marrow, spleen and thymus express α-defensins [28]. HNPs are endogenously expressed as inactive precursor pro-protein (proHNPs) that are proteolytically cleaved to generate the mature peptide. Intestinal proHNPs are processed by serine protease trypsin in humans [29] and matrix metalloprotease in mice [30]. However, a recent study has demonstrated that processing of neutrophil proHNPs may be independent of serine proteases [31], which indicates that the mechanisms underlying the posttranslational processing of HNPs are not completely elucidated and that the activity of other classes of proteases need to be examined in this context.

β-defensins are primarily secreted by epithelial cells, however, immune cells, such as monocytes, macrophages and dendritic cells, also express these peptides. β-defensins are expressed in mucosal secretions of gastrointestinal, urogenital and respiratory tracts. More than 20 potential β-defensin homologues have been described based on human genome analyses, of which hBD1-3 are most well characterized. Similar to HNPs, β-defensins are also expressed as a pro-peptide that is subjected to proteolytic cleavage to release the mature peptide. However, the proteases involved in the processing of β-defensins are not completely defined [32]. A recent study using computational analyses has demonstrated that β-defensins differ from α-defensins in the pattern of cysteine pairing and spacing, as well as having a shorter pro-peptide [33]. Despite the high degree of similarity in tertiary structures, β-defensins exhibit poor sequence similarities, suggesting that their bioactivity maybe independent of the primary amino acid sequences [34].

θ-defensins are macrocyclic peptides expressed in leukocytes and the bone marrow of nonhuman primates, such as macaques and baboons, and have not yet been conclusively identified either in humans or in other higher primates such as gorillas and chimpanzees [35,36,37]. θ-defensins exist in humans as a pseudogene due to the presence of a premature stop codon. Therefore, these peptides are called rare defensins with cyclic structures. The most well characterized θ-defensins are from rhesus macaque, and the rhesus θ-defensin 1 is the most abundant isoform in macaque granulocytes [38]. Furthermore, there are also CHDPs that lack typical secondary structure but are able to endorse novel folds, which include bovine indolicidin and porcine tritrpticin [26,27,39].

As mentioned above, research in the last three decades has demonstrated that CHDPs elicit a wide range of biological functions including an influence on the host’s immune responses for the resolution of infections and inflammation. CHDPs affect both innate and adaptive immunity, playing a critical role in immune homeostasis. CHDPs contribute to both immune activation and immune regulation, which is dependent on cellular composition, environmental stimuli and the kinetics of the inflammatory response. In this review, we will focus on the immune functions influenced by mammalian CHDPs.

## 2. Immune Activation Mediated by CHDPs

Innate immune response is the first line of host defense in response to infections. Microbial components, such as lipopolysaccharides (LPS), interact with host pattern recognition receptors such as toll-like receptors (TLRs). This results in the activation of multiple intracellular signaling networks and the production of numerous effector molecules, including CHDPs, that orchestrate host immune responses [40]. Accumulating evidence suggests that released CHDPs in response to an infectious challenge contribute to the enhancement of innate immune processes by inducing classical pro-inflammatory responses, such as recruitment of leukocytes to the site of infection, generation of reactive oxygen species and aiding phagocytosis, which collectively facilitate resolution of infections. In this context, one of the primary immune activating functions of CHDPs is to facilitate recruitment of leukocytes to the site of infections, either by direct chemoattractant properties or by mediating the induction of chemokines [10]. CHDPs, e.g., LL-37, HNP-1, HNP-2, hBD-1 and hBD-2 can directly promote chemotaxis of neutrophils, monocytes, eosinophils, dendritic cells (DCs) and T-lymphocytes to the site of infection (reviewed in [10]). In addition, CHDPs such as LL-37 and β-defensins (hBD-2 and hBD-3) at physiological concentrations can stimulate the production of various chemokines, e.g., Gro-α/CXCL1, IL-8/CXCL8, MCP-1/CCL2, MIP-1/CCL4, RANTES/CCL5 and MIP-3α/CCL20 [10,41,42]. Furthermore, CHDPs increase the expression of certain chemokine receptors to facilitate chemokine activity. For example, LL-37 induces the expression of chemokine receptors CCR2, CXCR4 and IL-8RB in macrophages, and hBD-1 and hBD-2 induce the expression of CCR6 on immature dendritic cells (iDCs) and memory T-cells [14,43,44]. Hence, one of the major anti-infective functions of CHDPs is to facilitate chemokine induction and activity. Other innate immune functions of CHDPs that facilitate the resolution of infections include the following; (i) the ability to mediate novel caspase-dependent anti-apoptotic activities to prolong the life-span of neutrophils. For example, LL-37 and hBD3 induce the expression of anti-apoptotic protein BcL-xl and inhibit caspase-3 activity resulting in the suppression of neutrophil apoptosis, and consequent increase of phagocytosis [45]. However, in vivo it remains to be determined how this activity changes the kinetics of regulation of consequent inflammation; (ii) CHDPs facilitate the formation of neutrophil extracellular traps (NETs) and mast cell derived extracellular traps (MCETs), which are extracellular DNA networks that can entrap pathogens and facilitate better microbial clearance [46]; (iii) recent studies have also demonstrated that CHDPs promote autophagy in macrophages thereby aiding intracellular killing and the clearance of bacterial infections [47,48]. The process of autophagy destroys phagosomes that harbor intracellular bacteria, such as *Mycobacterium tuberculosis*. CHDPs, in particular LL-37, have been shown to up-regulate the expression of autophagy related genes, and induce the formation of the autophagolysosome, to promote clearance of intracellular bacteria [47,48]. Overall, CHDPs exhibit multiple modes of action that influence the host immune system to resolve infections.

Signaling studies that have explored the mechanisms underlying the immune activating functions of CHDPs have demonstrated that these peptides influence key signaling pathways, in immune cells and structural cells, to promote downstream innate immune gene expression. For example, both LL-37 and β-defensins stimulate the activity of mitogen-activated protein kinases (MAPK) such as p38 and p44/42 in monocytes, mast cells, and keratinocytes [49,50,51,52]. LL-37 also activates signal transducer and activator of transcription 3 (STAT3) resulting in transactivation of epidermal growth factor receptor (EGFR) in keratinocytes [53]. Both cathelicidins and defensins also activate other fundamental signaling networks such as NF-κB, phosphatidylionositol-3 kinase (PI3K), activator protein (AP)-1 and Janus kinase (JAK) pathways [14,44,52,54]. The biological consequences of activation of MAPKs in conjunction with other signaling cascades, such as NF-κB, include the biosynthesis of pro-inflammatory cytokines, generation of reactive oxygen species, and activation of immune cells types, together orchestrating innate immunity and antimicrobial responses [55]. Despite many studies focused on delineating the signaling aspects, there are limited studies that define the direct interacting protein partners or the interactome of CHDPs. In a previous study, we demonstrated that cathelidicin LL-37 can modulate cellular responses in monocytes and activate p38 MAPK via direct interaction with glyceraldehyde-3-phosphate dehydrogenase (GAPDH) [56]. A recent in silico analysis of proteins known to interact with LL-37 showed that the human cathelicidin has more than a 1000 first order interacting protein partners [7]. This highlights the requirement of comprehensive Systems-level studies for understanding the interacting protein networks that are the basis for the immunological functions of CHDPs. In summary, the immune activating functions of CHDPs mediate the functions of a complex network of downstream signaling cascades that are responsible for effective defense mechanisms against invading pathogenic challenge.

## 3. Immune Regulation Mediated by CHDPs

The paradox in the bioactivity of CHDPs is the dual ability to mediate both pro- and anti-inflammatory responses. It is conceivable that these opposing responses are elicited by CHDPs with different kinetics; initial innate immune activating functions in response to pathogenic stimuli to elicit pro-inflammatory responses required to resolve infections, followed by the regulation of the inflammatory cascade to prevent ‘runaway’ chronic inflammation. However, the temporal dynamics of CHDP activities that are involved in regulating the inflammatory cascade are not completely understood. Nevertheless, research since the early 2000s has shown that one of the most defining roles of CHDPs (e.g., LL-37, hBD-2 and BAMP-28) is the ability to selectively control pathogen-induced inflammation [57,58,59]. Initial studies in this area suggested that the ability of CHDPs such as LL-37 to control bacterial endotoxin-induced inflammation is by direct binding to anionic LPS, thereby blocking LPS binding to LPS-binding protein, and consequently dampening immune activation by pattern recognition receptors [60]. However, further studies have demonstrated that CHDPs, such as cathelicidins human LL-37 and murine CRAMP, mediate anti-endotoxin and anti-inflammatory effects independent of LPS binding [3,41]. For example, cathelicidins intervene in innate immune signaling, particularly in the TLR-to-NFκB pathway [41,61], and α-defensin HNP-1 inhibits protein translation of pro-inflammatory cytokines in macrophages [62]. The anti-inflammatory role of CHDPs is also substantiated by observations that cathelicidin knockout mice exhibit increased inflammatory responses when compared to wild type [63]. Furthermore, we have clearly demonstrated that the human cathelicidin LL-37 suppresses downstream pro-inflammatory responses induced in the presence of a chronic inflammatory cytokine IL-32, and that it induces anti-inflammatory cytokines independent of microbial agonists [52]. These studies suggest that CHDPs exhibit immune regulatory activities that are independent of direct binding to endotoxin to control inflammation in the presence or absence of microbial agonists. It is therefore not surprising that some of the CHDPs and their derivative peptides were demonstrated to mitigate inflammatory responses in chronic diseases, such as ulcerative colitis, arthritis and autoimmune encephalomyelitis [64,65,66,67].

Another paradoxical mechanism that may be attributed to immune modulation by CHDPs is the ability to influence cell death processes. For example, CHDPs can induce apoptosis via an increase of intracellular reactive oxygen species (ROS) [68], and induce autophagy in macrophages during infectious challenge (described above; [47,48]). In contrast, CHDPs can inhibit apoptosis of neutrophils, thus prolonging the life-span of neutrophils to enhance bacterial clearance [45,69]. CHDPs can also inhibit lipid peroxidation mechanisms in oxidative stress thus resulting in the control of excessive ROS [68]. Overall, CHDPs play a critical role in maintaining the balance between cell death, inflammation and ROS production to restore tissue homeostasis (reviewed in [68]). Despite increasing evidence of the immune-regulatory functions of CHDPs, the precise role of these molecules in chronic inflammation has not been completely elucidated, as CHDPs can be both effectors and regulators in chronic inflammatory diseases [70]. Overall, these recent findings comprehensively demonstrate the wide range of functions mediated by CHDPs to regulate inflammation while maintaining the host immune responses that combat pathogenic infections.

## 4. CHDPs Bridge Innate and Adaptive Immunity

CHDPs within an inflammatory environment functionally co-operate with other innate immune cytokines such as IL-1β, IL-4 or GM-CSF to influence macrophage maturation and dendritic cell differentiation, and enhance antigen uptake and phagocytic activity by antigen presenting cells [71,72,73]. CHDPs promote recruitment of immature dendritic cells (iDCs) to the site of infections and within the tumor microenvironment [73,74,75]. CHDPs interact with several receptors on iDCs to influence dendritic cell maturation and differentiation [76]. Furthermore, CHDPs can promote the recruitment and migration of T-lymphocytes, and typically skew the polarization of antigen-specific adaptive immunity toward a T-helper (Th)-1 cell response [72,77]. These studies suggest that CHDPs function as a vital cog in bridging innate and adaptive responses, and that these peptides influence the initiation, polarization and amplification of specific adaptive immune responses. Consistent with this, several in vivo studies have demonstrated the adjuvant-like activity of CHDPs. For example, human defensins co-administered with antigens mimic adjuvants to activate humoral responses in mice causing increased production of antigen-specific serum antibodies [78]. A recent study has also demonstrated that oral administration of the cathelicidin peptide LL-37 elicits T-cell dependent antigen-specific antibody mucosal responses primarily via a Th17-skewed pathway, by interacting with receptors on M cells [79]. Studies effectively using synthetic derivatives of CHDPs as adjuvants in vaccine formulations have corroborated the role of these peptides in the adaptive immune response [80,81,82]. Taken together, these studies indicate that CHDPs function at the interface of innate and adaptive immune responses and play a critical role in promoting initiation, polarization and amplification of adaptive immunity.

## 5. Conclusions

Immune responses constitute a very complex network of opposing pro- and anti-inflammatory mediators, meticulously regulated for optimal immune homeostasis. CHDPs are immunomodulatory molecules that play a role in both immune activation and regulation, depending on the cellular microenvironment and exogenous or endogenous stimuli. The overall consequence of CHDP function is the control of inflammation along with maintenance of the immune responses required for resolution of infections. CHDPs also play a critical role in the crosstalk between innate and adaptive immunity, influencing the initiation and polarization of antigen-specific adaptive responses. Despite an increasing body of literature on the functions of CHDPs in immunity, there are significant gaps in knowledge. For example, the roles of CHDPs in chronic inflammation and autoimmunity remain elusive. Moreover, the underlying mechanisms governed by differential interactions with different interacting protein partners, which result in the diverse functions of CHDPs in immunity, have to be completely elucidated.
